# Association of IL-18 gene polymorphisms with clinical aspects of hyperlipidemia in middle-aged and early people in the community

**DOI:** 10.7150/ijms.90341

**Published:** 2024-04-22

**Authors:** Po-Chun Chen, Wei-Hung Chen, Li-Ming Lien, Chia-Chi Chou, Chia-Chia Chao, Chyi-Huey Bai

**Affiliations:** 1 School of Life Science, National Taiwan Normal University, Taipei, Taiwan.; 2 Translational medicine center, Shin-Kong Wu Ho-Su Memorial Hospital, Taipei City, Taiwan.; 3 Department of Medical Research, China Medical University Hospital, China Medical University, Taichung, Taiwan.; 4 Department of Neurology, Shin Kong Wu Ho-Su Memorial Hospital, Taipei 111045, Taiwan.; 5 School of Medicine, College of Medicine, Taipei Medical University, Taipei, Taiwan.; 6 Department of Neurology, Shin Kong Wu Ho-Su Memorial Hospital, Taipei, Taiwan.; 7 Institute of Epidemiology and Preventive Medicine, National Taiwan University, Taipei 100025, Taiwan.; 8 School of Medicine, Chang Gung University, Taoyuan 33302, Taiwan.; 9 Department of Internal Medicine, Chang Gung Memorial Hospital, Keelung 204201, Taiwan.; 10 Department of Respiratory Therapy, Fu Jen Catholic University, New Taipei City, Taiwan.; 11 Department of Public Health, School of Medicine, College of Medicine, Taipei Medical University, Taipei, Taiwan; 12 School of Public Health, College of Public Health, Taipei Medical University, Taipei, Taiwan.

## Abstract

Hyperlipidemia is notorious for causing coronary artery disease (CAD). IL-18 is a proinflammtory cytokine that contributes to the pathogenesis of CAD. Previous reports have revealed that genetic polymorphism of IL-18 is associated with its expression level as well as the susceptibility to CAD. In the present study, we aim to investigate the relationship between IL-18 single nucleotide polymorphisms (SNPs) and hyperlipidemia in the Han Chinese population in Taiwan. A total of 580 participants older than 30 were recruited from the community. We collected the demographics, self-reported disease histories, and lifestyles. We also assessed the levels of lipid profiles including total cholesterol (CHOL), triglyceride, low-density lipoprotein cholesterol (LDL-C) and high-density lipoprotein cholesterol. Two SNPs, rs3882891C/A (intron 5) and rs1946518A/C (promoter -607) of IL-18 were elucidated by polymerase chain reaction-restriction fragment length polymorphism (PCR-RFLP) methods. Our results revealed that rs3882891 AA was associated with lower risk of hypercholesterolemia, higher CHOL and LDL-C in subjects (p=0.003, p=0.000 and p=0.005 separately), and rs1946518 CC was associated with hypercholesterolemia, higher CHOL and LDL-C as well (p=0.021, p=0.003 and p=0.001 separately)

Furthermore, both SNPs were associated with IL-18 expression level, which was examined by Genotype-Tissue Expression (GTEx) Portal (p=0.042 and 0.016 separately). Finally, the haplotype of IL-18 was subsequently arranged in the order of rs3882891 and rs1946518. The result revealed that the AC haplotype of 2 IL-18 SNPs was also associated with lower risk of hypercholesterolemia, lower levels of CHOL and LDL-C (p=0.01, p=0.001 and 0.003). The current study is the first to report the association between IL-18 SNPs and hyperlipidemia in the Chinese Han population.

## Introduction

Dyslipidemia is notorious for elevated levels of blood cholesterol (CHOL), low-density lipoprotein cholesterol (LDL-C), or triglyceride (TG), while the decreased level of high-density lipoprotein cholesterol (HDL-C) 1. Dyslipidemia is one of the major risk factors related to coronary artery disease (CAD) 2, regarding to activate inflammatory responses caused by oxidation of LDL-C and very low density lipoprotein cholesterol (VLDL-C) 3, 4. Genetic factor such as single nucleotide polymorphism (SNP) is reported to have critical roles in dyslipidemia 5.

The pathogenic mechanisms of CAD include endothelium dysfunction, lipid peroxidation, and inflammation 6. With regard to the pivotal role of inflammation in hyperlipidemia-related CAD 7, 8, investigation of the association between SNPs of inflammatory genes and hyperlipidemia is urgent.

The interleukin-18 (IL-18) was first characterized as an interferon-γ-inducing cytokine, with pleiotropic proinflammatory activities which maintain immune responses 9. Previous study shows that deletion of IL-18 develops dyslipidemia in mice 10. Moreover, elevated expression of IL-18 is associated with hypertension, type 2 diabetes, metabolic syndrome, as well as the formation of atherosclerotic plaques 11-14, the development and pathogenesis of CAD 15, 16.

The genetic association of IL-18 and its effect on the progression of CAD has been investigated previously. Several studies have discussed the role of genetic polymorphisms on the IL-18 expression level, which contributes to the prognosis of CAD 17-20. Furthermore, rs1946518 polymorphism which located on IL-18 promoter region is associated with expression regulation 20. Previous report showed that rs3882891 polymorphism is associated with an increased risk of acute myocardial infarction 21. However, the association between these two IL-18 genetic polymorphism and dyslipidemia, which is the major cause of CAD, is rarely defined. Therefore, we evaluated two IL-18 SNPs (rs3882891C/A; NC_000011.10:112144037:C:A and rs1946518A/C; NC_000011.10:112164734:A:C) as candidate biomarkers for susceptibility to dyslipidemia. Our finding here reveals an obvious association between alternative alleles of rs3882891 and rs1946518 polymorphisms, and lower risk of hypercholesterolemia, lower circulating CHOL and LDL-C levels.

## Materials and Methods

### Subjects

The current study was a community-based prospective cohort study aimed to investigate individuals' the cardiovascular disease and cerebrovascular risk factors. The method of enrolled participant collection and the exclusion criteria were provided elsewhere 22. All participants lived in Wen Shan and Shi Lin districts near Shin Kong Wu Ho-Su Memorial Hospital and Wan Fang Hospital. Only Chinese Han were included in this study. Exclusion criteria were listed: age ≤ 30, incomplete questionnaire, prior history of cancer or chronic kidney disease, refusal to blood collection or duplex ultrasound measured. Finally, 580 participants were included. All subjects signed a consent form to the original study; no names were published in the results. The study protocol received approval from the institutional ethics committee, and the investigations were conducted according to the Declaration of Helsinki for experiments involving humans. (IRB number: 96E086).

### Data collection

Well-trained nurses interviewed all subjects using a structured questionnaire. Subjects' blood was collected after at least 8 hours of fasting. The samples were centrifuged for 15 minutes at 3000 g, separated into plasma, serum, and buffy coat tubes, and stored at 80°C. Technologists assessed CHOL, TG, LDL-C, and HDL-C levels in a standardized laboratory. Hypercholesterolemia is CHOL>250mg/dL or LDL-C>160mg/dL. Hypertriglyceridemia is defined as the level of TG>200mg/dL. Higher CHOL, TG, and LDL-C are defined separately as >200mg/dL, >150mg/dL, and >130mg/dL. Lower HDL-C is defined as <40mg/dL.

### Genotyping

The genomic DNA was purified from the buffy coat by using the salting-out method as described in the previous study 23. Genomic DNA was extracted from the buffy coat using the EasyPure® Genomic DNA Kit (Cat.No.GB100; Bioman Scientific Co., LTD, Taiwan), following the manufacturer's guidelines. The DNA was dissolved in TE buffer (10 mM Tris, pH 7.8, 1 mM EDTA) and preserved at -20°C until further analysis was performed. Genotyping of SNPs was performed by polymerase chain reaction-restriction fragment length polymorphism (PCR-RFLP) methods.

The information of primers, restriction enzymes and PCR conditions for detecting IL-18 SNPs rs1946518A/C and rs3882891C/A was provided in the Supplementary information. Sequence validation and duplicate genotyping from 5% to 10% of blinded samples were done. In order to confirm accuracy, 60 samples were chosen at random and subjected to blind duplicate genotyping. The bases of an additional 30 samples were sequenced and compared to the results of genotyping.

### Bioinformatics analysis

We conducted an analysis of genotype-phenotype correlation using data from the Genotype-Tissue Expression (GTEx) Portal (https://www.gtexportal.org/home/) 24, with a significance threshold set at p < 0.05. The following analysis investigated the relationship between rs1946518A/C and rs3882891C/A and IL-18 expression performance in whole blood utilizing information extracted from the GTEx database.

### Statistical analysis

For continuous covariates, mean±standard deviation (SD) was showed, and number(percentage) was showed for categorical data. Student's t-test was used to compare continuous variables between two groups. The genotype distribution of each SNP was analyzed for Hardy-Weinberg equilibrium and confirmed by χ2 analysis. χ2 tests were performed to compare the categorical variables between the two groups. Adjusted odds ratios (AORs) with 95% confidence intervals (CIs) were conducted by multiple logistic regression analyses that controlled for age and gender. Haplotype frequencies were analyzed using Haploview, and statistical analysis of the haplotype structure was performed, as summarized in the manuscript 25. All analyses were performed by using SPSS statistical software (version 25). Statistical significance was set at p < 0.05.

## Results

All study subjects were Chinese Han. The total 580 participants were enrolled in the current study. The average age of subjects was approximately 60 years old. About 56% of subjects were male and more than 80% of the subjects had dyslipidemia including hypercholesterolemia (CHOL>250mg/dL or LDL-C>160mg/dL) or and hypertriglyceridemia (TG>200mg/dL). After adjustment of age and gender, the AOR to the risk of lipid abnormalities in IL-18 genotypes are provided in Tables 2. All genotypic frequencies were in Hardy-Weinberang equilibrium (p > 0.05). In both control and case groups, the highest distribution frequencies for rs3882891 and rs1946518 were heterozygous for CA and AC, respectively. The recessive models of rs3882891 showed lower risks of hypercholesterolemia, high CHOL and high LDL-C in participants with higher significant variation (AOR=0.431, CI=0.226-0.823, p=0.011; AOR=0.411, CI=0.263-0.644, p=0.000; AOR=0.602, CI=0.397-0.913, p=0.017). Furthermore, the AA genotype of rs3882891 was associated with the lower risks of hypercholesterolemia, high CHOL and high LDL-C in participants (AOR=0.314, CI=0.148-0.667, p=0.003; AOR=0.337, CI=0.198-0.574, p=0.000; AOR=0.488, CI=0.295-0.807, p=0.005 separately) (Table 2).The recessive models of rs1946518 were significantly associated with lower CHOL level and LDL-C (AOR=0.512, CI=0.325-0.807, p=0.004; AOR=0.544, CI=0.350-0.847, p=0.007) and CC genotype of rs1946518 showed similar phenomenon, with lower risk to hypercholesterolemia (AOR=0.421, CI=0.202-0.877, p=0.021), high CHOL (AOR=0.435, CI=0.254-0.747, p=0.003) and high LDL-C levels (AOR=0.409, CI=0.242-0.692, p=0.001) (Table 2). In our investigation, the AA genotype of rs3882891 and CC genotype of rs1946518 shows lower risk of developing lipid abnormalities.

**Figure 1:**
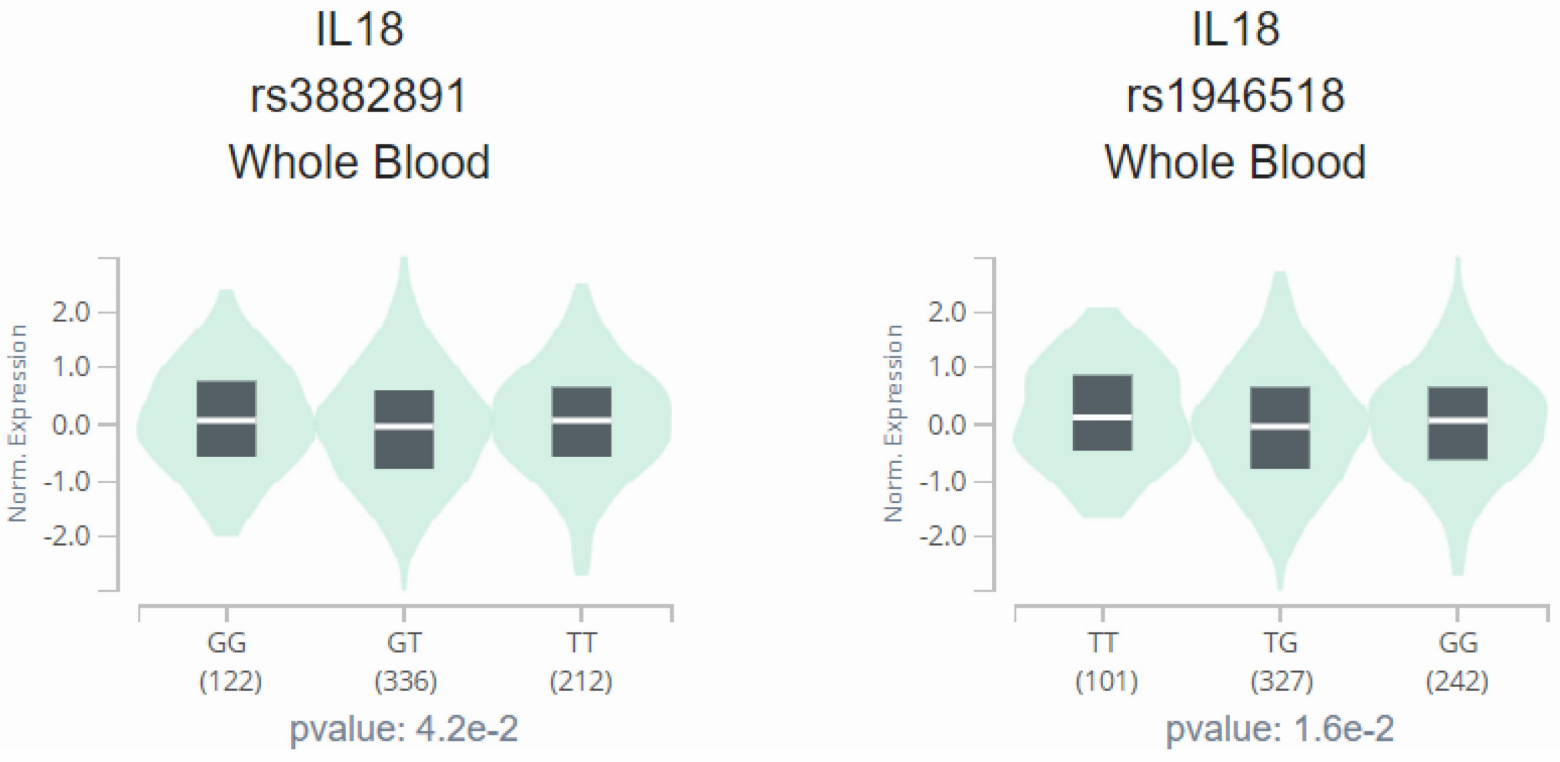
IL-18 displays a significant eQTL association with rs3882891 (A) and rs1946518 (B) genotypes in lung tissues (GTEx data set).

The analysis results of Genotype-Tissue Expression (GTEx) Portal based on IL-18 SNPs (rs1946518 and rs3882891) indicated the inverse relationship between both SNPs and IL-18 expression, and the individuals with the AA genotype of rs3882891 and CC genotype of rs1946518 showed lower expression of IL-18 than reference genotypes in a whole blood sample (p=0.042 and 0.016, Figure 1).

**Figure 2:**
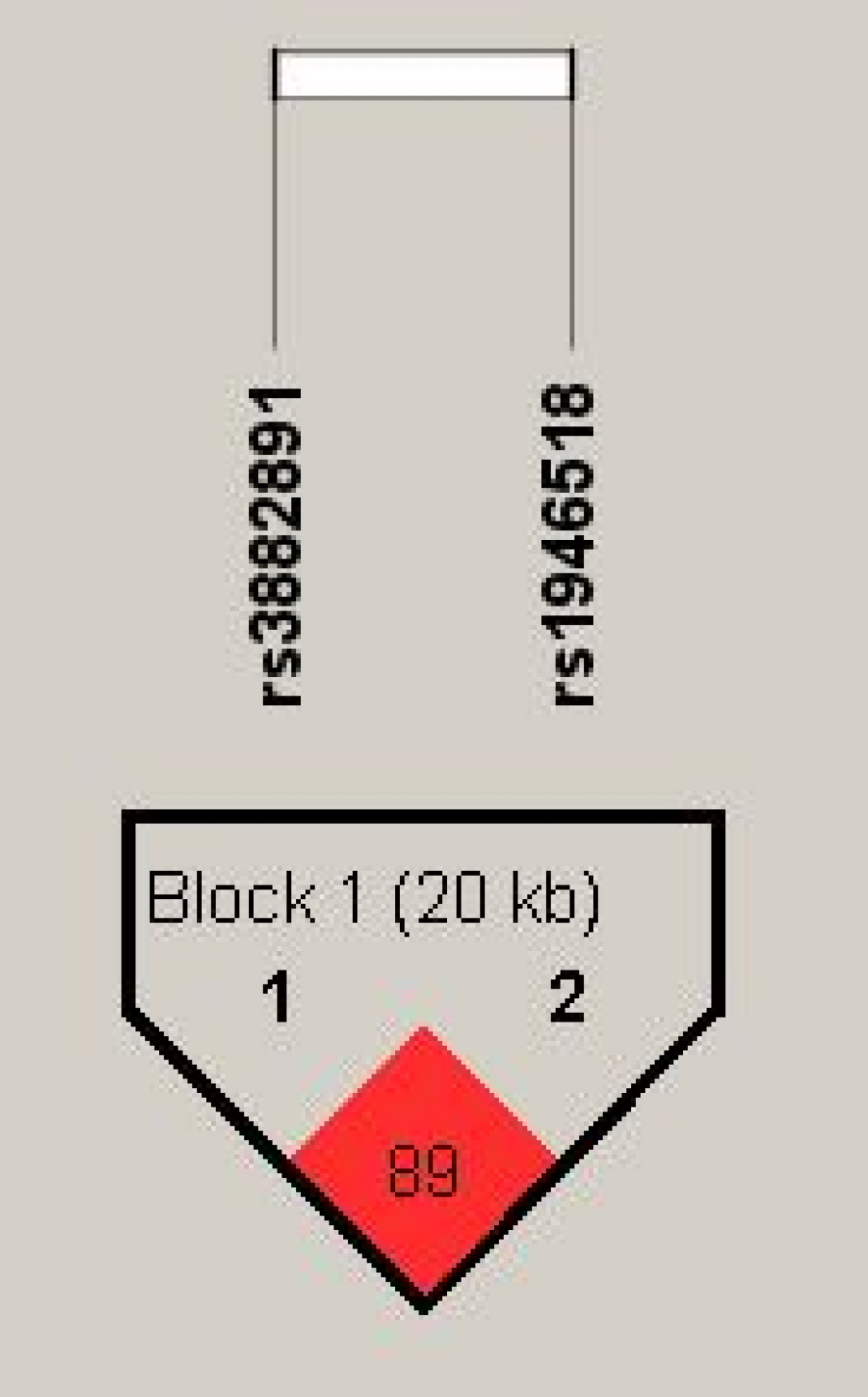
Displaying the pairwise linkage disequilibrium (LD) patterns of IL-18. A schematic illustration of IL-18 is provided, highlighting the positions of SNP polymorphisms and depicting the D' values for pairwise linkage disequilibrium. The D' values for each SNP are visually- represented using a grayscale, with white denoting low D' and darker shades indicating high D'.

Haplotype analysis based on the genotype data in the order of rs3882891 and rs1946518, revealing these two SNPs were in high linkage disequilibrium (89%) in halpoblock (Figure 2). Both minor alleles of rs3882891 and rs1946518 which compose the AC haplotype has lower risk of developing lipid abnormalities, such as hypercholesterolemia (OR=0.439, CI=0.231-0.835, p=0.01), higher CHOL (OR=0.665, CI=0.521-0.849, p=0.001), and higher LDL-C (OR=0.696, CI=0.546-0.887, p = 0.003) (Table 3).

## Discussion

The main findings of this work were the obvious associations between genotypes AA and CC of two IL-18 SNPs (rs3882891 and rs1946518) and lower risk of hypercholesterolemia, lower circulating CHOL and LDL-C levels, which may contribute to the progression of CAD. The data also found that subjects who carry genotypic variants show lower circulating IL-18 levels than wild type.

Genetic variation of IL-18 has been proven to associate with or even affect the circulating level of IL-18 in previous reports. The previous study evaluated 5 SNPs located in promoter and 5'UTR regions of IL-18, and the data showed that in response to stimulation, haplotypes 1 (G-C-G-T-C) and 3 (T-A-G-T-C) could enhance promoter transcriptional activity than haplotype 2 (T-A-C-G-T) 20. Another study investigated IL-18 expression after lipopolysaccharide (LPS) exposure, and the results indicated that the subjects who carried the -137 GG genotype produced more IL-18 than the heterozygous genotype 26. The IL-18-5848 T>C was associated with IL-18 levels in coronary artery bypass graft (CABG) patients, not only in the baseline but also 6 hours postoperative 27. Here, we found that both IL-18 SNPs (rs3882891 and rs1946518) were associated with IL-18 expression by utilizing the online Genotype-Tissue Expression (GTEx) Portal. However, the previous study has found no significant association of IL-18 rs1946518 and IL-18 serum level in CAD patients 28. This could contribute to ethnic differences which lead to a different distribution of the allele frequency.

Although the previous report has provided evidence that IL-18 rs1946518 polymorphism was associated with susceptibility to CAD in East Asians 29, there is no relevant report that investigates the association between IL-18 rs1946518 and dyslipidemia, one of the major causes involved in the pathogenesis of CAD. In accordance with previous findings 29, our results indicate that the genotypic variants of IL-18 rs1946518 is associated with lower circulating CHOL and LDL-C levels. Furthermore, previous study shows that rs1946518 polymorphism, located within the IL-18 promoter region, is implicated in the regulation of IL-18 expression 20. This finding proposes a mechanism by which rs1946518 polymorphism is related to gene regulation of IL-18, which in turn affects lipid levels in circulation and thus contributing to the progression of CAD. However, the previous study reveals that IL-18 knockout (IL-18 -/-) mice develop hypercholesterolemia, hyper-high-density-lipoprotein cholesterolemia, and hypertriglyceridemia while aging 10. With regard to the pleiotropic property of IL-18 in biological function, complete knockout of IL-18 gene expression may elicit some adverse effects. However, the molecular mechanism involved in lipid metabolism by IL-18 expression should be examined in the future.

The subjects who carry the AA genotypic variant of rs3882891 show a lower prevalence of circulating CHOL and LDL-C levels. Furthermore, the rs3882891 CC genetic variant shows lower expression of IL-18. A previous study indicated a correlation between the rs3882891 polymorphism and an elevated risk of acute myocardial infarction 21. As high levels of IL-18 have been correlated with an elevated risk of developing cardiovascular diseases, including acute myocardial infarction, and are considered a therapeutic target 30, the impact of the rs3882891 polymorphism on IL-18 expression, as well as its association with dyslipidemia, need to be elucidated in future studies.

Several limitations in this study should be addressed. Although subjects were enrolled from communities in Taiwan, only individuals of Chinese Han were included in this study and the sample size was small exclusion criteria for participant enrollment. Moreover, the present work provided observation results, and the molecular mechanism should be delineated to confirm our findings here.

## Supplementary Material

Supplementary information and figures.

## Figures and Tables

**Table 1:** Demographical characteristics in 580 subjects in current study.

General Characteristics and Serum Lipid Levels of the Subjects	
**All subjects (N=580)**	**Mean±SD or N (%)**
**Age (yrs)**	60.83± 11.468
**Gender**	
Male	326 (56.2%)
Female	254 (43.8%)
**Hypertension**	**SBP>140, DBP>90**
No	343 (59.1%)
Yes	237 (40.9%)
**Hypercholesterolemia**	**CHOL>250 or LDL-C>160**
No	483 (89.5%)
Yes	97 (10.5%)
**Hypertriglyceridemia**	**TG>200**
No	482 (83.1%)
Yes	98 (16.9%)
**CHOL_subgroup**	**>200**
No	351 (60.5%)
Yes	229 (39.5%)
**TG_subgroup**	**>150**
No	321 (55.3%)
Yes	259 (44.7%)
**LDL-C_subgroup**	**>130**
No	340 (58.6%)
Yes	240 (41.4%)
**HDL-C_subgroup**	**<40**
No	382 (65.9%)
Yes	198 (34.1%)
**SBP**	132.20 ± 22.559
**DBP**	82.31 ± 40.190
**CHOL**	183.81 ± 59.285
**TRIG**	152.09 ± 84.310
**LDL**	125.52 ± 32.724
**HDL**	47.59 ± 14.663

**Table 2:** Odds ratio (OR), adjusted odds ratio (AOR), and related 95% confidence interval (CI) of hyperlipidemia associated with IL-18 genotypic frequencies.

			All subjects (N=580) n (%)					
			**Hypercholesterolemia (T-chol>250mg/dL, LDL-C>160mg/dL)**					
**Gene (SNP)**	**Genetic model**		**No (N=483)**	**Yes (N=97)**	**OR (95% CI)**	***p* values**	**AOR (95% CI)**	***p* values**
**rs3882891**	Dominant	CC	106 (22%)	31 (31.6%)	1		1	
		CA+AA	376 (78%)	67 (68.4%)	0.609 (0.378-0.982)*	**0.041**	0.581 (0.357-0.946)*	**0.029**
	Recessive	CC+CA	363 (75.3%)	86 (87.8%)	1		1	
		AA	119 (24.7%)	12 (12.2%)	0.426 (0.225-0.806)*	**0.007**	0.431 (0.226-0.823)*	**0.011**
	Additive	CC	106 (22%)	31 (31.6%)	1		1	
		CA	257 (53.3%)	55 (56.1%)	0.732 (0.446-1.200)	0.215	0.703 (0.425-1.162)	0.169
		AA	119 (24.7%)	12 (12.2%)	0.345 (0.169-0.706)*	**0.003**	0.314 (0.148-0.667)*	**0.003**
**rs1946518**	Dominant	AA	108 (22.4%)	29 (29.6%)	1		1	
		AC+CC	374 (77.6%)	69 (70.4%)	0.687 (0.424-1.114)	0.127	0.626 (0.381-1.028)	0.064
	Recessive	AA+AC	379 (78.6%)	85 (86.7%)	1		1	
		CC	103 (21.4%)	13 (13.3%)	0.563 (0.302-1.049)	0.067	0.564 (0.300-1.061)	0.076
	Additive	AA	108 (22.4%)	29 (29.6%)	1		1	
		AC	271 (56.2%)	56 (57.1%)	0.770 (0.466-1.270)	0.304	0.710 (0.426-1.184)	0.189
		CC	103 (21.4%)	13 (13.3%)	0.470 (0.232-0.954)*	**0.034**	0.421 (0.202-0.877)*	**0.021**
								
			**Hypertriglyceridemia (TG>200mg/dL)**					
**Gene (SNP)**	**Genetic model**		**No (N=465)**	**Yes (N=115)**	**OR (95% CI)**	***p* values**	**AOR (95% CI)**	***p* values**
**rs3882891**	Dominant	CC	107 (23%)	30 (26.1%)	1		1	
		CA+AA	358 (77%)	85 (73.9%)	0.847 (0.530-1.353)	0.487	0.832 (0.519-1.35)	0.446
	Recessive	CC+CA	356 (76.6%)	93 (80.9%)	1		1	
		AA	109 (23.4%)	22 (19.1%)	0.773 (0.463-1.289)	0.322	0.796 (0.475-1.333)	0.385
	Additive	CC	109 (22.6%)	28 (28.9%)	1		1	
		CA	261 (54%)	51 (52.6%)	0.902 (0.553-1.473)	0.681	0.877 (0.535-1.438)	0.603
		AA	113 (23.4%)	18 (18.6%)	0.72 (0.391-1.327)	0.291	0.727 (0.393-1.346)	0.31
**rs1946518**	Dominant	AA	107 (23%)	30 (26.1%)	1		1	
		AC+CC	358 (77%)	85 (73.9%)	0.847 (0.530-1.353)	0.487	0.809 (0.504-1.300)	0.382
	Recessive	AA+AC	366 (78.7%)	98 (85.2%)	1		1	
		CC	99 (21.3%)	17 (14.8%)	0.641 (0.366-1.124)	0.118	0.651 (0.370-1.145)	0.136
	Additive	AA	107 (23%)	30 (26.1%)	1		1	
		AC	259 (55.7%)	68 (59.1%)	0.936 (0.577-1.521)	0.791	0.889 (0.544-1.451)	0.637
		CC	99 (21.3%)	17 (14.8%)	0.612 (0.318-1.179)	0.14	0.599 (0.31-1.159)	0.128
								
			**Higher CHOL (>200 mg/dL)**					
**Gene (SNP)**	**Genetic model**		**No (N=351)**	**Yes (N=229)**	**OR (95% CI)**	***p* values**	**AOR (95% CI)**	***p* values**
**rs3882891**	Dominant	CC	71 (20.2%)	66 (28.8%)	1		1	
		CA+AA	280 (79.8%)	163 (71.2%)	0.626 (0.425-0.922)*	**0.017**	0.605 (0.407-0.898)*	**0.013**
	Recessive	CC+CA	252 (71.8%)	197 (86.0%)	1		1	
		AA	99 (28.2%)	32 (14.0%)	0.413 (0.266-0.642)*	**0.000**	0.411 (0.263-0.644)*	**0.000**
	Additive	CC	71 (20.2%)	66 (28.8%)	1		1	
		CA	181 (51.6%)	131 (57.2%)	0.779 (0.520-1.166)	0.224	0.751 (0.497-1.134)	0.174
		AA	99 (28.2%)	32 (14%)	0.348 (0.207-0.585)*	**0.000**	0.337 (0.198-0.574)*	**0.000**
**rs1946518**	Dominant	AA	75 (21.4%)	62 (27.1%)	1		1	
		AC+CC	276 (78.6%)	167 (72.9%)	0.732 (0.497-1.078)	0.114	0.685 (0.461-1.019)	0.062
	Recessive	AA+AC	267 (76.1%)	197 (86.0%)	1		1	
		CC	84 (23.9%)	32 (14.0%)	0.516 (0.330-0.807)*	**0.003**	0.512 (0.325-0.807)*	**0.004**
	Additive	AA	75 (21.4%)	62 (27.1%)	1		1	
		AC	192 (54.7%)	135 (59%)	0.851 (0.569-1.271)	0.43	0.794 (0.526-1.198)	0.272
		CC	84 (23.9%)	32 (14%)	0.461 (0.272-0.781)*	**0.004**	0.435 (0.254-0.747)*	**0.003**
								
			**Higher TG (>150 mg/dL)**					
**Gene (SNP)**	**Genetic model**		**No (N=321)**	**Yes (N=259)**	**OR (95% CI)**	***p* values**	**AOR (95% CI)**	***p* values**
**rs3882891**	Dominant	CC	73 (22.7%)	64 (24.7%)	1		1	
		CA+AA	248 (77.3%)	195 (75.3%)	0.897 (0.611-1.317)	0.579	0.897 (0.610-1.318)	0.579
	Recessive	CC+CA	247 (76.9%)	202 (78.0%)	1		1	
		AA	74 (23.1%)	57 (22.0%)	0.942 (0.636-1.394)	0.765	0.944 (0.637-1.399)	0.775
	Additive	CC	73 (22.7%)	64 (24.7%)	1		1	
		CA	174 (54.2%)	138 (53.3%)	0.905 (0.6041-1.354)	0.626	0.904 (0.603-1.354)	0.623
		AA	74 (23.1%)	57 (22%)	0.879 (0.543-1.422)	0.598	0.880 (0.543-1.426)	0.605
**rs1946518**	Dominant	AA	75 (23.4%)	62 (23.9%)	1		1	
		AC+CC	246 (76.6%)	197 (76.1%)	0.969 (0.659-1.424)	0.872	0.971 (0.659-1.428)	0.879
	Recessive	AA+AC	258 (80.4%)	206 (79.5%)	1		1	
		CC	63 (19.6%)	53 (20.5%)	1.054 (0.700-1.585)	0.802	1.056 (0.701-1.590)	0.795
	Additive	AA	75 (23.4%)	62 (23.9%)	1		1	
		AC	183 (57%)	144 (55.6%)	0.952 (0.637-1.422)	0.81	0.953 (0.637-1.426)	0.815
		CC	63 (19.6%)	53 (20.5%)	1.018 (0.619-1.672)	0.945	1.021 (0.621-1.679)	0.935
			**Higher LDL-C (>130mg/dL)**					
**Gene (SNP)**	**Genetic model**		**No (N=340)**	**Yes (N=240)**	**OR (95% CI)**	***p* values**	**AOR (95% CI)**	***p* values**
**rs3882891**	Dominant	CC	70 (20.6%)	67 (27.9%)	1		1	
		CA+AA	270 (79.4%)	173 (72.1%)	0.669 (0.455-0.984)*	**0.041**	0.656 (0.444-0.970)*	**0.034**
	Recessive	CC+CA	251 (73.8%)	198 (82.5%)	1		1	
		AA	89 (26.2%)	42 (17.5%)	0.598 (0.396-0.903)*	**0.014**	0.602 (0.397-0.913)*	**0.017**
	Additive	CC	70 (20.6%)	67 (27.9%)	1		1	
		CA	181 (53.2%)	131 (54.6%)	0.756 (0.505-1.132)	0.174	0.738 (0.491-1.111)	0.146
		AA	89 (26.2%)	42 (17.5%)	0.493 (0.3-0.81)*	**0.005**	0.488 (0.295-0.807)*	**0.005**
**rs1946518**	Dominant	AA	68 (20%)	69 (28.7%)	1		1	
		AC+CC	272 (80%)	171 (71.3%)	0.620 (0.421-0.911)*	**0.015**	0.589 (0.398-0.872)*	**0.008**
	Recessive	AA+AC	259 (76.2%)	205 (85.4%)	1		1	
		CC	81 (23.8%)	35 (14.6%)	0.546 (0.353-0.845)*	**0.006**	0.544 (0.350-0.847)*	**0.007**
	Additive	AA	68 (20%)	69 (28.7%)	1		1	
		AC	191 (56.2%)	136 (56.7%)	0.702 (0.47-1.048)	0.083	0.666 (0.443-1)	0.05
		CC	81 (23.8%)	35 (14.6%)	0.426 (0.253-0.716)*	**0.001**	0.409 (0.242-0.692)*	**0.001**
			**Lower HDL-C (<40 mg/dL)**					
**Gene (SNP)**	**Genetic model**		**No (N=382)**	**Yes (N=198)**	**OR (95% CI)**	***p* values**	**AOR (95% CI)**	***p* values**
**rs3882891**	Dominant	CC	87 (22.8%)	50 (25.3%)	1		1	
		CA+AA	295 (77.2%)	148 (74.7%)	0.873 (0.585-1.302)	0.505	0.868 (0.570-1.320)	0.508
	Recessive	CC+CA	288 (75.4%)	161 (81.3%)	1		1	
		AA	94 (24.6%)	37 (18.7%)	0.704 (0.460-1.079)	0.106	0.693 (0.444-1.080)	0.105
	Additive	CC	87 (22.8%)	50 (25.3%)	1		1	
		CA	201 (52.6%)	111 (56.1%)	0.961 (0.633-1.459)	0.852	0.96 (0.619-1.49)	0.857
		AA	94 (24.6%)	37 (18.7%)	0.685 (0.409-1.147)	0.149	0.674 (0.393-1.154)	0.15
**rs1946518**	Dominant	AA	85 (22.3%)	52 (26.3%)	1		1	
		AC+CC	297 (77.7%)	146 (73.7%)	0.804 (0.540-1.196)	0.281	0.805 (0.530-1.223)	0.309
	Recessive	AA+AC	301 (78.8%)	163 (82.3%)	1		1	
		CC	81 (21.2%)	35 (17.7%)	0.798 (0.514-1.239)	0.314	0.793 (0.502-1.254)	0.322
	Additive	AA	85 (22.3%)	52 (26.3%)	1		1	
		AC	216 (56.5%)	111 (56.1%)	0.840 (0.555-1.271)	0.409	0.844 (0.546-1.304)	0.444
		CC	81 (21.2%)	35 (17.7%)	0.706 (0.418-1.195)	0.194	0.705 (0.407-1.22)	0.211

**Table 3:** Odds ratio (OR) and 95% confidence interval (CI) of hyperlipidemia associated with IL-18 rs3882891/rs1946518 haplotype frequencies.

All subjects (N=1160) n (%)				
	Hypercholesterolemia (T-chol>250mg/dL, LDL-C>160mg/dL)			
**Haplotype**	**No**	**Yes**	**OR (95% CI)**	***p* values**
**rs3882891, rs1946518**				
**CA 2**	344 (43.7%)	81 (82.7%)	1	
**AC 3**	116 (24.1%)	12 (12.2%)	0.439 (0.231-0.835)*	**0.01**
**CC 1**	19 (3.9%)	5 (5.1%)	0.895 (0.324-2.468)	0.83
**AA 4**	3 (0.6%)	0 (0%)	0.809 (0.773-0.848)	0.401
	** Higher CHOL (>200 mg/dL)**			
**Haplotype**	**No**	**Yes**	**OR (95% CI)**	***p* values**
**rs3882891, rs1946518**				
**CA 2**	307 (43.7%)	251 (54.8%)	1	
**AC 3**	344 (49.0%)	187 (40.8%)	0.665 (0.521-0.849)*	**0.001**
**CC 1**	16 (2.3%)	12 (2.6%)	1.090 (0.506-2.347)	0.825
**AA 4**	35 (5%)	8 (1.7%)	0.280 (0.127-0.614)*	**0.001**
	**Higher LDL-C (>130mg/dL)**			
**Haplotype**	**No**	**Yes**	**OR (95% CI)**	***p* values**
**rs3882891, rs1946518**				
**CA 2**	302 (44.4%)	256 (53.3%)	1	
**AC 3**	334 (49.1%)	197 (41.0%)	0.696 (0.546-0.887)*	**0.003**
**CC 1**	19 (2.8%)	9 (1.9%)	1.790 (0.796-4.024)	0.154
**AA 4**	25 (3.7%)	18 (3.8%)	0.849 (0.453-1.592)	0.61
