# Supplementary material for: Association of IL-18 gene polymorphisms with clinical aspects of hyperlipidemia in middle-aged and early people in the community
Source: Int J Med Sci. 2024 Apr 22;21(6):1064–71. doi: 10.7150/ijms.90341 (PMC11103393; doi:10.7150/ijms.90341)
Supplement: Supplementary file 1 — Supplementary information and figures. [file ijmsv21p1064s1.pdf]

Supplementary information

The conditions for PCR-RFLP of the SNPs of IL-18 were listed as follows. For restriction enzyme reaction, all related products were purchased from Fermentas (Waltham, MA, USA). The PCR reaction was performed with PCR machine (LifePro Thermal cycler, LifePro TC-96/G/H(b)A; BIOER TECHNOLOGY Corporation; Hangzhou, China). PCR reaction was performed in the 30 µl mixture containing 20 µl doubledistilled water (DDW), 1.5 µl forward and reverse primers, 2 µl dNTP (2.5 mM), 1 µl Taq polymerase, 3 µl 10x PCR buffer and 1 µl DNA genome.

The restriction enzyme digestion was carried out in 10 µl mixture containing 3 µl of PCR products, 5.5 µl of DDW, 1 µl of digestion buffer, and 0.5 µl restriction enzymes, followed by an incubation period of 20 mins at 37°C. Subsequently, the digested products were subjected to electrophoresis on a 3% agarose gel, stained with ethidium bromide (EtBr), and visualized under UV illumination. The 100 bp DNA Ladder (DL-100BP; Bento Lab; London, UK) was used as reference of fragments' size.

rs3882891C/A (intron 5)

Primers: 5'-ATCGTCTTTAGCCGTGAATG-3'

5'-TAACCTCATTCAGGACTTC C-3'

PCR conditions: 94°C for 5 min→ 35 cycles of 94°C for 30s, 57.1°C for 30s, 72°C for 30s.→ 72°C for 5 min. The amplicon was 400 base-pair (bp).

Restriction enzyme: *FspBI*

The presence of two bands of 243bp and 157bp showed the existence of A variant (figure S1).

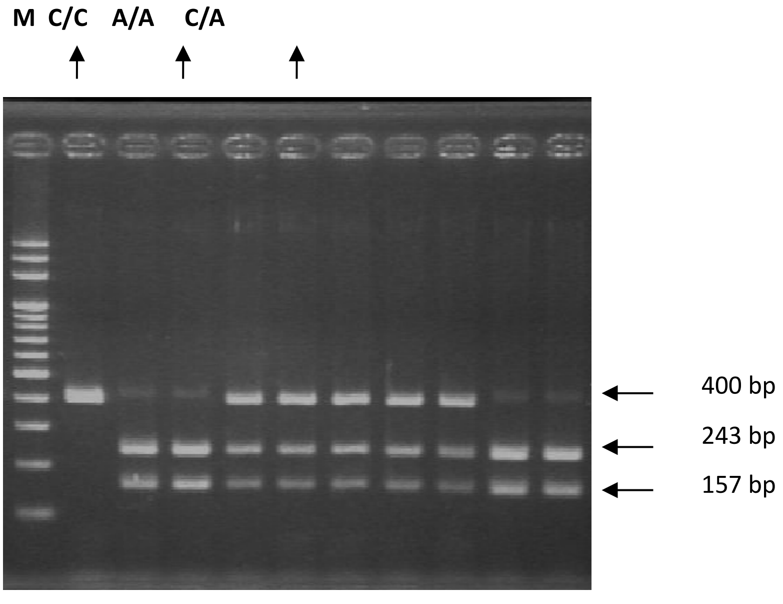

Figure S1. PCR-RFLP analysis of SNP rs3882891A/C of the IL-18 gene. The amplified product of the SNP rs3882891A/C was digested with *FspBI*.

rs1946518A/C (promoter -607)

Primers: 5'-AGATTTACTTTTCAGTGGAACAGGAGTCC-3'

5'-GCCCTCTTAC CTGAATTTTGGTATCCCTC-3'

PCR conditions: 94°C for 5 min→ 35 cycles of 94°C for 30s, 58°C for 30s, 72°C for 30s.→ 72°C for 5 min. The amplicon was 171 base-pair (bp).

Restriction enzyme: *MseI*

The presence of two bands of 101bp and 70bp showed the existence of A variant (figure S2).

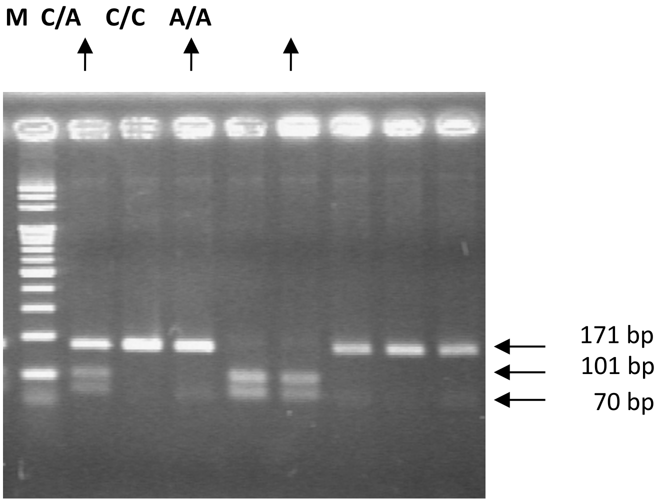

Figure S2. PCR-RFLP analysis of SNP rs1946518A/C of the IL-18 gene. The amplified product of the SNP rs3882891A/C was digested with *MseI*.
